# Comparison of the complete mitochondrial genome of *Phyllophorus liuwutiensis* (Echinodermata: Holothuroidea: Phyllophoridae) to that of other sea cucumbers

**DOI:** 10.1002/2211-5463.12914

**Published:** 2020-07-17

**Authors:** Fuyuan Yang, Chen Zhou, Ngoc Tuan Tran, Zaiqiao Sun, Jianshao Wu, Hui Ge, Zhen Lu, Chenhui Zhong, Zhihuang Zhu, Qiuhua Yang, Qi Lin

**Affiliations:** ^1^ Key Laboratory of Cultivation and High‐value Utilization of Marine Organisms in Fujian Province Fisheries Research Institute of Fujian Xiamen China; ^2^ College of Fisheries and Life Science Shanghai Ocean University China; ^3^ Institute of Marine Sciences Guangdong Provincial Key Laboratory of Marine Biotechnology Shantou University China

**Keywords:** mitochondrial genome, *Phyllophorus liuwutiensis*, sea cucumber, sequence analysis, structure characteristic

## Abstract

Sea cucumber species are abundant (>1400 species) and widely distributed globally. mtDNA sequencing is frequently used to identify the phylogenetic and evolutionary relationships among species. However, there are no reports on the mitochondrial genome of *Phyllophorus liuwutiensis*. Here, we performed mtDNA sequencing of *P. liuwutiensis* to examine its phylogenetic relationships with other echinoderms. Its mitochondrial genome (15 969 bp) contains 37 coding genes, including 13 protein‐coding genes, 22 tRNA genes and 2 rRNA genes. Except for one protein‐coding gene (*nad6*) and five tRNA genes encoded on the negative strand, all other genes were encoded on the positive strand. The mitochondrial bases of *P. liuwutiensis* were composed of 29.55% T, 22.16% C, 35.64% A and 12.64% G. The putative control region was 703 bp in length. Seven overlapping regions (1–10 bp) were found. The noncoding region between the genes ranged from 1 to 130 bp in length. One putative control region has been found in the *P. liuwutiensis* mitogenome. All of the tRNA genes were predicted to fold into a cloverleaf structure. In addition, we compared the gene arrangements of six echinoderms, revealing that the gene order of *P. liuwutiensis* was a new arrangement.

## Graphical abstract

In this paper, we analyzed the mitochondrial genome of *Phyllophorus liuwutiensis*. We examined genome composition, base composition, codon usage of protein‐coding genes and gene arrangement. In addition, we compared the gene arrangements of six echinoderms, revealing that the gene order of *P. liuwutiensis* was a new arrangement.

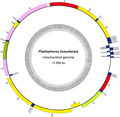

**Abbreviations**
CR: control region
PCG: protein‐coding gene
RSCU: relative synonymous codon usage

Sea cucumbers belong to the phylum Echinodermata, which are an important food source for human, particularly in some parts of Asia [1]. Sea cucumbers are nocturnal feeding species, hiding by day in coral reef rocks or sand [2]. The species of sea cucumbers are abundant and widely distributed throughout the world. There are more than 1400 species of sea cucumbers in the world, which are distributed in shallow sea areas, trenches and other marine environments [3]. Despite the variety and wide distribution of sea cucumbers, the phylogenetic and evolutionary relationships of sea cucumbers remain largely unknown.

In metazoan animals, the mitochondrial genomes are characterized by exposed circular double‐stranded DNA molecules [4]. It has been found that most mitochondrial genomes are self‐replicated and inherited in the maternal line. mtDNA sequencing is frequently used to identify the phylogenetic and evolutionary relationships among species [5, 6, 7]. Because of its rapid evolution and the lack of genetic recombination, mtDNA can provide important information about rearrangement laws and phylogenetic relationship [8]. The mtDNA contains 37 genes, including 13 protein‐coding genes (PCGs), 2 rRNA genes and 22 tRNA genes [9, 10]. At present, many studies have investigated the phylogeny and evolution of echinoderms. Mu *et al*. [3] have explored the adaptation of sea cucumber in a deep‐sea environment based on the research of the mtDNA. Fan *et al*. [11] have found a new sequence of genes in the mitochondrial genome of the *Stichopus horrens*. However, no study has been carried out on the mitochondrial genome of *Phyllophorus liuwutiensis*. Therefore, we, in this study, aimed to clarify the structure and composition of the mitochondrial genome of *P. liuwutiensis*, and its phylogeny and evolution were also discussed.

The sea cucumbers, *P. liuwutiensis* (Holothuroidea: Dendrochirotida: Phyllophoridae: Phyllophorus), are popularly distributed in the intertidal sandy bottom. In China, *P. liuwutiensis* is distributed in only the two provinces of Fujian and Guangdong. *P. liuwutiensis* has a thin, cylinder‐shaped body with a length of 90–200 mm and a diameter of 10–28 mm [12]. The whole body of *P. liuwutiensis* is covered by tube feet. It has a polian vesicle with a length of about 40 mm. The body wall bone is termed as a table, and the chassis has a round or irregular shape. Moreover, it has a hole in the center, and there are eight (or more than eight) other holes in the peripheral area. The rim of the chassis is an undulating shape, and the diameter of the disc ranges from 50 to 80 μm [12].

## Materials and methods

### Sample collection and identification

All animal handling procedures were reviewed and approved by the ethics committee of the ‘Regulations for the Administration of Affairs Concerning Experimental Animals’. The Institutional Animal Care and Use Committee of Fisheries Research Institute of Fujian, China, approved the experiments.

*P. liuwutiensis* were collected in Huandao Road, Xiamen City, Fujian Province, China. The back muscle of sea cucumber was clipped, fixed by anhydrous ethanol and stored at −20 °C [11]. The back wall bone fragment was used to identify the species using a scanning electron microscope [13]. The body wall of the sea cucumber was treated with a 10% NaClO solution for 1–2 min. Then the white precipitate was washed with distilled water four times. The samples were dried and then gold‐plated by an ion sputtering device. Finally, the samples were examined using a scanning electron microscope [11].

### DNA extraction

DNA was extracted from 30 mg back muscle tissue using the TIANamp Marine Animals DNA Kit (Tiangen Biochemical Technology, Beijing, Co. Ltd., Beijing, China) according to the manufacturer's instructions. The obtained DNA was stored at −20 °C prior to further analysis.

### PCR amplification and sequencing

The complete mitochondrial nucleotide sequence of *P. liuwutiensis* was obtained by general and long‐range PCR amplification using the specific primers (Table 1). The primers were designed to match the generally conserved regions of target mtDNA, and the short fragments of *cox*1, *cox*2, *atp*6, *cox* *3*, *nad* *4*, *cytb*, *12S*, *nad* *1* and *16S* were amplified. In brief, the amplifications were carried out with 40 cycles at a melting temperature of 94 °C for 30 s, an annealing temperature of 50 °C for 30 s and an extension temperature of 72°C for 1 min per 1 kb. The final MgCl_2_ concentration in the reaction was 2.0 mmol·L^−1^. PCR products were cloned into a pMD18‐T vector (Takara, Beijing, China) and then sequenced using the dideoxynucleotide procedure by ABI 3730 automatic sequencer. Sequences were assembled by dnastar software [14] and manually adjusted to generate the complete sequence of mtDNA.

**Table 1:** Primers used for amplification of complete mitochondrial genome of *P. liuwutiensis*.

Fragment no.	Gene or region	Primer name	Sequence (5′–3′)	Length (bp)
F1	*cox1*	MIF1	CGAACAGAACTAGCCCAACC	1411
		MIR1	CTTTGAATGTGTGGTGAGATGG	
F2	*cox1‐cox2*	MIF2	TCCATCTCCTCCATAGGATC	708
		MIR2	GCAATAGAATATTGATAAGAGG	
F3	*cox2*	MIF3	GAGCACAAATTGGATTACAAG	595
		MIR3	GCTCCACAGATTTCTGAGCA	
F4	*cox2‐atp6*	MIF4	GAGTTAAGATGGATGGAGTTC	584
		MIR4	GAAGCTTTGTGGCTTCTAGG	
F5	*atp6*	MIF5	CGACACAATAGGATTTCTCC	607
		MIR5	GTAAGCTTGGATACATGCAAC	
F6	*atp6‐cox3*	MIF6	GCCACCTGAGTCCTTAGATC	322
		MIR6	CATCATCTTATAGCAGTTAGG	
F7	*cox3*	MIF7	GTTGATCAAAGACCATGACC	711
		MIR7	GACGTCAACGAAGTGTCAGT	
F8	*cox3‐nd4*	MIF8	CAACCTTCCTAACAGTATGTC	1404
		MIR8	TAGGGAGCCTAGGGCACAGAAG	
F9	*nad4*	MIF9	CCAAAGGCCCACGTAGAAGC	228
		MIR9	GTGGCCTACAGAAGAATATGC	
F10	*nad4‐cytb*	MIF10	CCTACTCCGACTCTTCCCTC	3446
		MIR10	CTTGATTTATGTAGGATCCA	
F11	*cytb*	MIF11	GCACTACACCGCTGACATAAC	880
		MIR11	AGGTTCTTCTACTGGTTGGC	
F12	*cytb‐12S*	MIF12	CCAATCATCCTCCTTTCGAC	646
		MIR12	GTATAGCGGGGTATCTAATCC	
F13	*12S*	MIF13	CACGTTAACCTTTAGCTAAAG	570
		MIR13	GGTACACCTACTTTGTTACG	
F14	*12S‐nad1*	MIF14	GTACCTCCTTAAAGAAATAAG	2206
		MIR14	GATCATAAAGCAATTGCTAAAG	
F15	*nad1*	MIF15	GTAGTTGGGCCATACGGATTAC	725
		MIR15	CGTGGGTAGGAGGCTCGTAC	
F16	*nad1‐16S*	MIF16	CTAGGCGGTAGAAGACCATTC	1955
		MIR16	CTTGGTTTTTGTTTATGTTTCC	
F17	*16S*	MIF17	CCTTTAGTAGACCTAAAAGC	863
		MIR17	GGTCCTTTCGTACTAAAGAAGG	
F18	*16S‐cox1*	MIF18	GTAACCAAAGGGTGCAGCAG	568
		MIR18	CAATCATTAGTGGAATTAGTC	

### Sequence analysis and gene annotation

After the quality proofing of the obtained fragments, the mtDNA sequences were manually assembled using dnastar v7.1 software [14]. First, the raw mtDNA sequences were imported into MITOS web servers to determine the approximate boundaries of genes. Exact positions of PCGs were found by searching open reading frames. All tRNA genes were identified using ARWEN, DOGMA and MITOS [15, 16, 17]. MEGAX was used to calculate the DNA base composition and codon preference of the mitochondrial genome of *P. liuwutiensis* [18]. Formula with GC‐skew = (G − C)/(G + C) and the AT‐skew = (A − T)/(A +T) were calculated for base's preferences [19].

### Phylogenetic analysis

The *P. liuwutiensis* and another 25 echinoderm mitogenomes (obtained from GenBank; https://www.ncbi.nlm.nih.gov/) were used for phylogenetic analysis. *Balanoglossus carnosus* (Enteropneusta) was rooted as the outgroup. Echinoderms were divided into five classes as follows: Holothuroidea, Echinoidea, Asteroidea, Ophiuroidea and Crinoidea [20]. Therefore, the species of these five classes were selected to construct the evolutionary tree, among which more sea cucumber species were selected to study the phylogeny and evolution of the *P. liuwutiensis*. The megax [18] was used to perform the alignment of 13 PCGs. A Bayesian approach using mrbayes 3.1.2 version [21, 22] was employed to analyze the aligned datasets and trees. Analyses had two parallel runs with four chains of each (three hot chains and one cold chain), which were carried out for 1 000 000 generations (sampling every 100 generations). After the first 1000 ‘burn in’ trees were discarded, the remaining 9000 sampled trees were used to estimate the 50% majority rule consensus tree and the Bayesian posterior probabilities.

## Results

### Identification of *P. liuwutiensis*

**Fig. 1:**
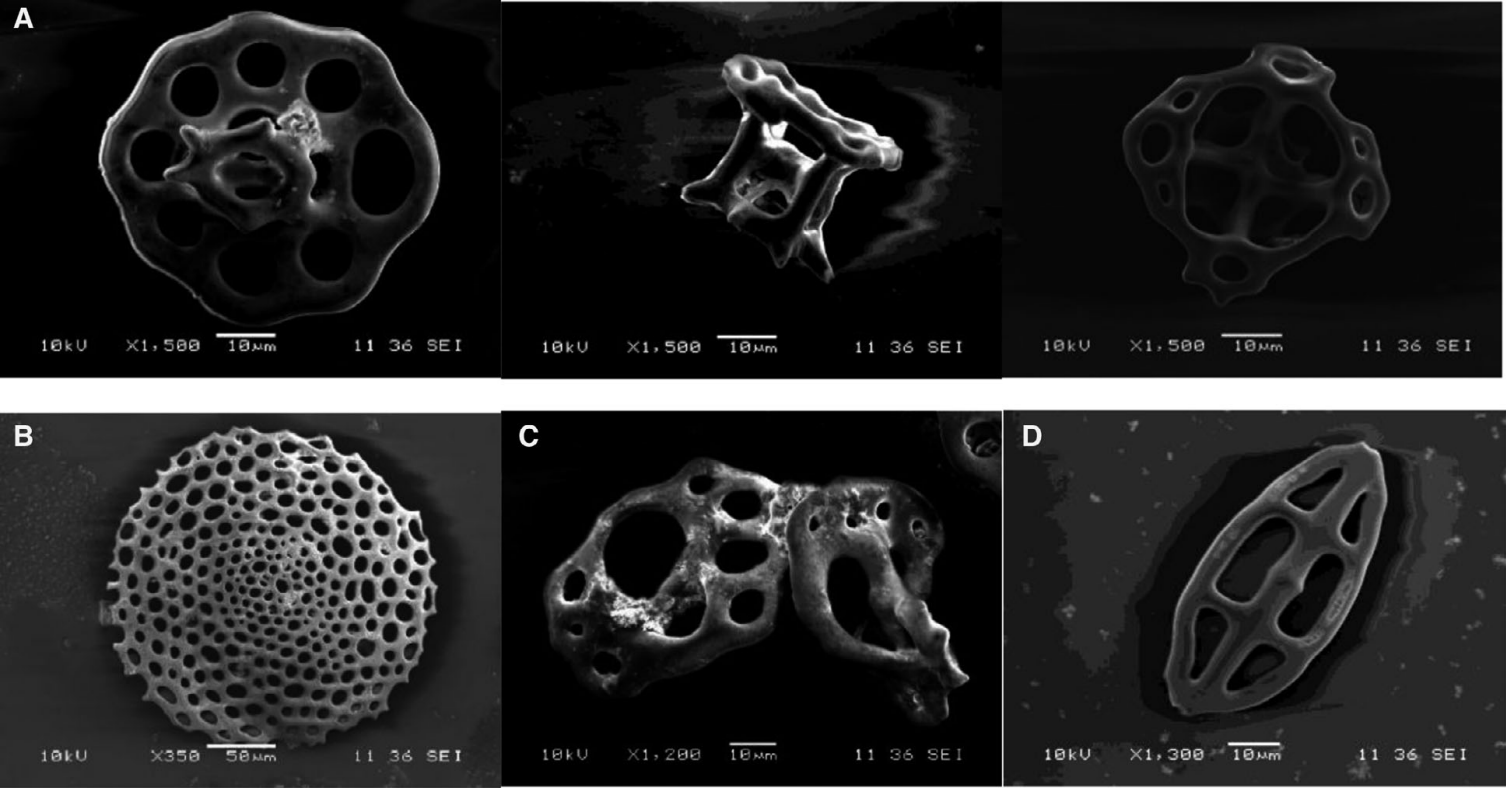
The dorsal wall of the *P. liuwutiensis*. Tabular form (A), plates of sea cucumber (B), type rosettes (C) and type buttons (D). Scale bars: 10 μm (A, C, D); 50 μm (B).

We found four types of table body in the body wall of the sea cucumber (Fig. 1), which was in accordance with a previous study [12]. Based on the morphological characteristics, the sea cucumber was identified to be *P. liuwutiensis*.

### Genome organization

**Fig. 2:**
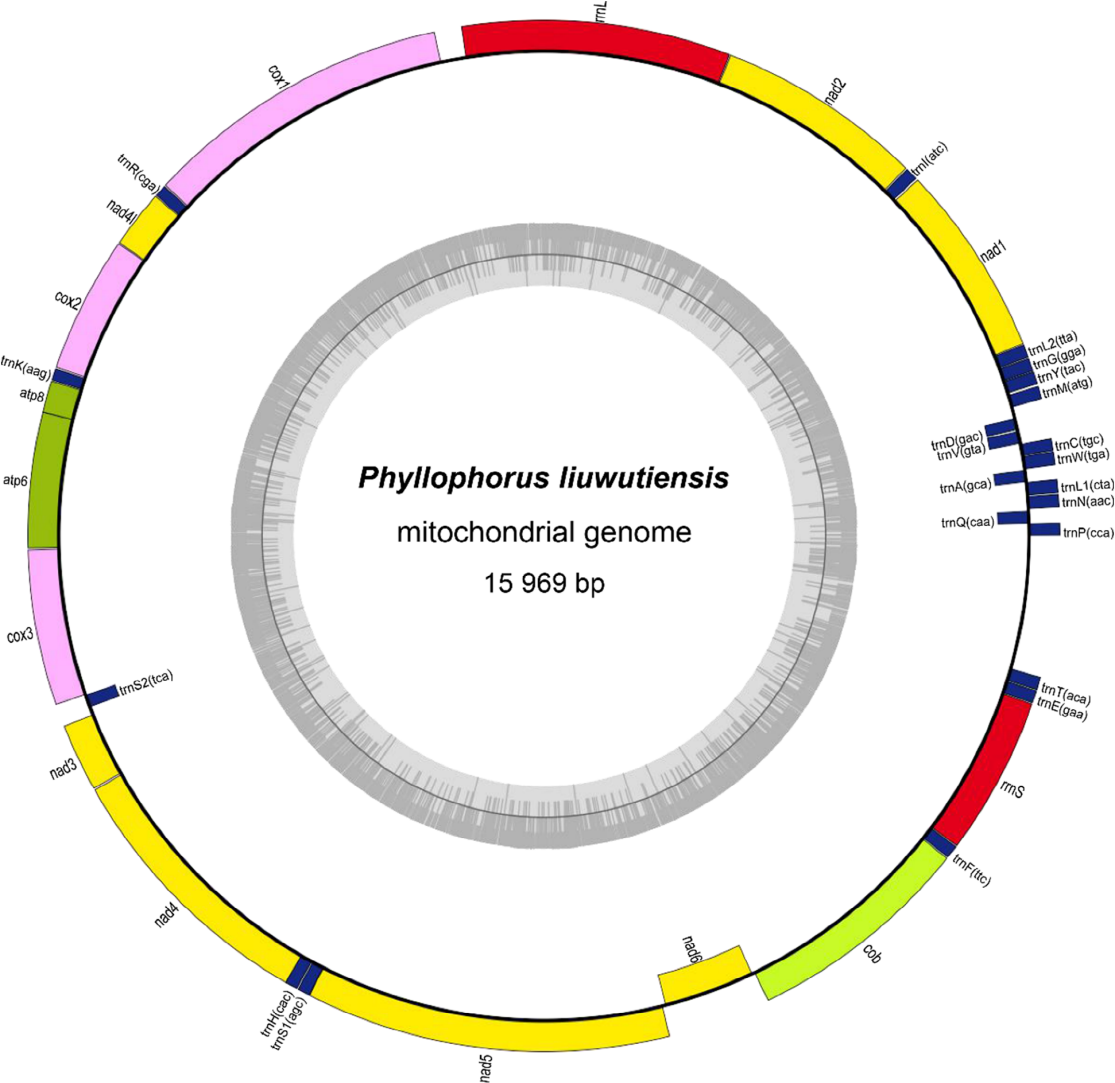
Gene map of the complete mitochondrial genome of *P. liuwutiensis*. Genes encoded on the positive and negative strands are shown outside and inside the circular gene map, respectively.

The complete mtDNA sequence of *P. liuwutiensis* was obtained by general and long‐range PCR. Similar to other deuterostomes, the mtDNA of *P. liuwutiensis* was a closed double‐stranded loop and composed of 15 969 bp (Fig. 2). The genome encoded 37 genes, including 13 PCGs, 2 rRNA genes and 22 tRNA genes. Among these genes, 31 genes were encoded on the positive strand, whereas others (such as *nad6*, *tRNA^ser(tga)^*, *tRNA^Gln^*, *tRNA^Ala^*, *tRNA^Val^* and *tRNA^Asp^*) were encoded on the negative strand. The base composition of the mtDNA of *P. liuwutiensis* is 29.55% T, 22.16% C, 35.64% A and 12.64% G.

### Base composition and AT/GC‐skew of mtDNA

Table 2 shows the base composition of *P. liuwutiensis* and 25 species of echinoderms. We also found that the contents of the bases varied among species, with a T content of 31.63% ranging from 25.24% (*Acanthaster planci*) to 46.46% (*Phanogenia gracilis*), a C content of 21.00% ranging from 11.44% (*P. gracilis*) to 27.95% (*A. planci*), an A content of 32.25% ranging from 25.82% (*P. gracilis*) to 35.82% (*Echinaster brasiliensis*) and a G content of 15.12% ranging from 10.65% (*Freyastera benthophila*) to 18.22% (*Strongylocentrotus droebachiensis*; Table 2). The length of the *P. liuwutiensis* mtDNA was shorter compared with other echinoderms. The higher content of base A (than T) and the lower content of base G (than C) were found in most echinoderms. The data (Table 2) showed that all GC skewness of the *P. liuwutiensis* mtDNA was negative, and all AT skewness was positive, indicating that the mtDNA of *P. liuwutiensis* had a preference for A and C.

**Table 2:** List of species used for species identification based on the whole mitochondrial genome and the analysis of the base composition of each species.

Species	T (%)	C (%)	A (%)	G (%)	A + T (%)	GC‐skew	AT‐skew
*P. liuwutiensis*	29.55	22.16	35.64	12.64	65.19	−0.273	0.093
*Amphiura digitula*	30.89	22.98	32.74	13.39	63.63	−0.263	0.029
*Apostichopus japonicus*	30.14	20.21	31.75	17.89	61.89	−0.061	0.026
*Parastichopus nigripunctatus*	30.14	20.15	31.68	18.03	61.82	−0.055	0.025
*Parastichopus parvimensis*	29.91	20.38	31.78	17.93	61.69	−0.064	0.030
*Parastichopus californicus*	29.87	20.57	31.53	18.04	61.40	−0.066	0.027
*Stichopus* sp. SF‐2010	29.29	23.76	30.95	16.01	60.24	−0.195	0.028
*Stichopus horrens*	29.31	23.72	30.80	16.17	60.11	−0.189	0.025
*Holothuria scabra*	27.07	24.34	32.67	15.91	59.74	−0.209	0.094
*Holothuria forskali*	30.82	21.41	31.40	16.37	62.22	−0.134	0.009
*Cucumaria miniata*	28.09	22.95	35.74	13.22	63.83	−0.269	0.120
*Benthodytes marianensis*	36.91	17.83	32.33	12.93	69.24	−0.160	−0.066
*Freyastera benthophila*	33.53	21.13	34.70	10.65	68.23	−0.330	0.017
*Astropecten polyacanthus*	31.51	22.44	32.50	13.55	64.01	−0.247	0.015
*Acanthaster planci*	25.24	27.95	31.10	15.71	56.34	−0.280	0.104
*Echinaster brasiliensis*	25.83	26.43	35.82	11.91	61.65	−0.379	0.162
*Heliocidaris crassispina*	27.86	24.37	31.03	16.74	58.89	−0.186	0.054
*Heterocentrotus mammillatus*	29.02	23.55	29.89	17.54	58.91	−0.146	0.015
*Strongylocentrotus droebachiensis*	30.18	22.80	28.80	18.22	58.98	−0.112	−0.023
*Hemicentrotus pulcherrimus*	30.58	22.43	29.19	17.79	59.77	−0.115	−0.023
*Florometra serratissima*	46.37	11.57	26.45	15.61	72.82	0.149	−0.274
*Phanogenia gracilis*	46.46	11.44	25.82	16.27	72.28	0.174	−0.286
*Ophiura lutkeni*	33.11	19.23	32.77	14.90	65.88	−0.127	−0.005

### Overlapping and noncoding regions

In this study, we identified seven overlapping regions of genes (*trnP/trnQ*, *trnL1/trnA*, *trnY/trnG*, *atp8/atp6*, *cox3/trnS2*, *nad4/trnH* and *trnS1/nad5*) in the complete genome of *P. liuwutiensis* (Table 3). The length of these overlaps varied from 1 to 10 bp, with the longest length between *nad4* and *trnH* (Table 3). Among the 20 noncoding regions, the length was 1–703 bp (Table 3). The longest noncoding sequence (703 bp; AT% = 69.30%) was found between *tRNA^thr^* and *tRNA^pro^*. Due to its location and AT richness, the noncoding sequence in *P. liuwutiensis* was identified as the putative control regions (CRs; Table 3), which were similar to that in the genomic studies of *Stichopus* sp. [23].

**Table 3:** Annotation of the mitochondrial genome of *P. liuwutiensis*.

Gene	Strand	Sequence location	Size (bp)	Start codon	Stop codon	Intergenic region
*trna‐pro*	+	1–66	66			−4
*trna‐gln*	−	63–132	70			6
*trna‐asn*	+	139–207	69			2
*trna‐leu(tag)*	+	210–282	73			−1
*trna‐ala*	−	282–348	67			1
*trna‐trp*	+	350–419	70			0
*trna‐cys*	+	420–484	65			1
*trna‐val*	−	486–555	70			4
*trna‐asp*	−	560–624	65			57
*trna‐met*	+	682–750	69			7
*trna‐tyr*	+	758–826	69			−1
*trna‐gly*	+	826–896	71			0
*trna‐leu(taa)*	+	897–967	71			0
*nad1*	+	968–1939	972	GTG	TAA	5
*trna‐ile*	+	1945–2012	68			2
*nad2*	+	2015–3055	1041	ATG	TAA	0
*16S*	+	3056–4403	1348			130
*cox1*	+	4534–6087	1554	ATG	TAA	1
*trna‐arg*	+	6089–6153	65			0
*nad4L*	+	6154–6450	297	ATG	TAA	0
*cox2*	+	6451–7140	690	ATG	TAA	2
*trna‐lys*	+	7143–7206	64			0
*atp8*	+	7207–7371	165	ATG	TAA	−7
*atp6*	+	7365–8048	684	ATG	TAA	2
*cox3*	+	8051–8833	783	ATG	TAA	−1
*trna‐ser(tga)*	−	8833–8903	71			39
*nad3*	+	8943–9287	345	ATG	TAA	3
*nad4*	+	9291–10 652	1362	ATG	TAG	−10
*trna‐his*	+	10 643–10 710	68			1
*trna‐ser(gct)*	+	10 712–10 778	67			−3
*nad5*	+	10 776–12 605	1830	TTG	TAA	13
*nad6*	−	12 619–13 107	489	CTA	CAT	8
*cytb*	+	13 116–14 255	1140	ATG	TAA	1
*trna‐phe*	+	14 257–14 327	71			0
*12S*	+	14 328–15 128	801			0
*trna‐glu*	+	15 129–15 196	68			0
*trna‐thr*	+	15 197–15 266	70			0
Putative CR	+	15 267–15 969	703			0

### PCGs and codon usage

The length of PCGs in the mtDNA of *P. liuwutiensis* was 11 352 bp. The longest (1830 bp) and shortest (165 bp) lengths were *nad5* and *atp8*, respectively (Table 3). The bases C and A were found to be predominant in most genes, while the base T was predominant in some genes (Table 4). Except for *nad6*, which was encoded on the negative strand, all PCGs were encoded on the positive strand (Table 3 and Fig. 2), which was similar to the results found in other sea cucumber species [3]. ATG was found to be the start codon in most PCGs, except for *nad1* (GTG as the start codon), *nad5* (TTG) and *nad6* (CTA), while TAA was the termination codon of most PCGs, excepting *nad4* (TAG as the start codon) and *nad6* (CAT; Table 3).

**Table 4:** The base composition and preference of mitochondrial gene in *P. liuwutiensis*.

Gene	T (%)	C (%)	A (%)	G (%)	G + C (%)	Total (bp)	GC‐skew	AT‐skew
PCGs	30.20	23.60	34.10	12.10	35.70	11 352	−0.322	0.061
*nad6*	18.40	22.90	50.51	8.18	31.08	489	−0.474	0.466
*nad5*	28.96	22.79	38.25	10.00	32.79	1830	−0.390	0.138
*nad4L*	35.35	23.91	32.32	8.42	32.32	297	−0.479	−0.045
*nad4*	29.59	25.11	35.17	10.13	35.24	1362	−0.425	0.086
*nad3*	34.20	23.77	30.14	11.88	35.65	345	−0.333	−0.063
*nad2*	34.49	21.13	33.14	11.24	32.37	1041	−0.306	−0.020
*nad1*	34.67	20.88	30.76	13.68	34.57	972	−0.208	−0.060
*cytb*	28.33	25.70	33.51	12.46	38.16	1140	−0.347	0.084
*cox3*	29.76	25.93	29.50	14.81	40.74	783	−0.273	−0.004
*cox2*	30.72	24.20	31.74	13.33	37.54	690	−0.290	0.016
*cox1*	29.99	23.42	30.24	16.34	39.77	1554	−0.178	0.004
*atp8*	23.64	16.36	49.09	10.91	27.27	165	−0.200	0.350
*atp6*	30.70	26.02	32.31	10.96	36.99	684	−0.407	0.026
*trna‐val*	32.86	21.43	30.00	15.71	37.14	70	−0.154	−0.045
*trna‐tyr*	27.54	15.94	37.68	18.84	34.78	69	0.083	0.156
*trna‐trp*	34.29	12.86	44.29	8.57	21.43	70	−0.200	0.127
*trna‐thr*	35.71	11.43	41.43	11.43	22.86	70	0.000	0.074
*trna‐ser*	29.58	26.76	29.58	14.08	40.85	71	−0.310	0
*trna‐ser(gct)*	31.34	17.91	28.36	22.39	40.30	67	0.111	−0.050
*trna‐pro*	36.36	9.09	36.36	18.18	27.27	66	0.333	0.000
*trna‐phe*	25.35	16.90	40.85	16.90	33.80	71	0	0.234
*trna‐met*	33.33	18.84	33.33	14.49	33.33	69	−0.130	0
*trna‐lys*	31.25	17.19	39.06	12.50	29.69	64	−0.158	0.111
*trna‐leu*	34.25	16.44	32.88	16.44	32.88	73	0.000	−0.020
*trna‐leu(taa)*	29.58	16.90	33.80	19.72	36.62	71	0.077	0.067
*trna‐ile*	32.35	19.12	32.35	16.18	35.29	68	−0.083	0
*trna‐his*	32.35	16.18	38.24	13.24	29.41	68	−0.100	0.083
*trna‐gly*	30.99	15.49	40.85	12.68	28.17	71	−0.100	0.137
*trna‐glu*	26.47	19.12	36.76	17.65	36.76	68	−0.040	0.163
*trna‐gln*	31.43	20.00	35.71	12.86	32.86	70	−0.217	0.064
*trna‐cys*	41.54	9.23	33.85	15.38	24.62	65	0.250	−0.102
*trna‐asp*	36.92	21.54	26.15	15.38	36.92	65	−0.167	−0.171
*trna‐asn*	24.64	17.39	37.68	20.29	37.68	69	0.077	0.209
*trna‐arg*	32.31	15.38	38.46	13.85	29.23	65	−0.053	0.087
*trna‐ala*	35.82	13.43	38.81	11.94	25.37	67	−0.059	0.040
*16S*	27.74	17.36	39.09	15.80	33.16	1348	−0.047	0.170
*12S*	21.22	21.72	41.57	15.48	37.20	801	−0.168	0.324
CRs	26.60	21.30	42.70	9.40	30.70	703	−0.388	0.232

**Fig. 3:**
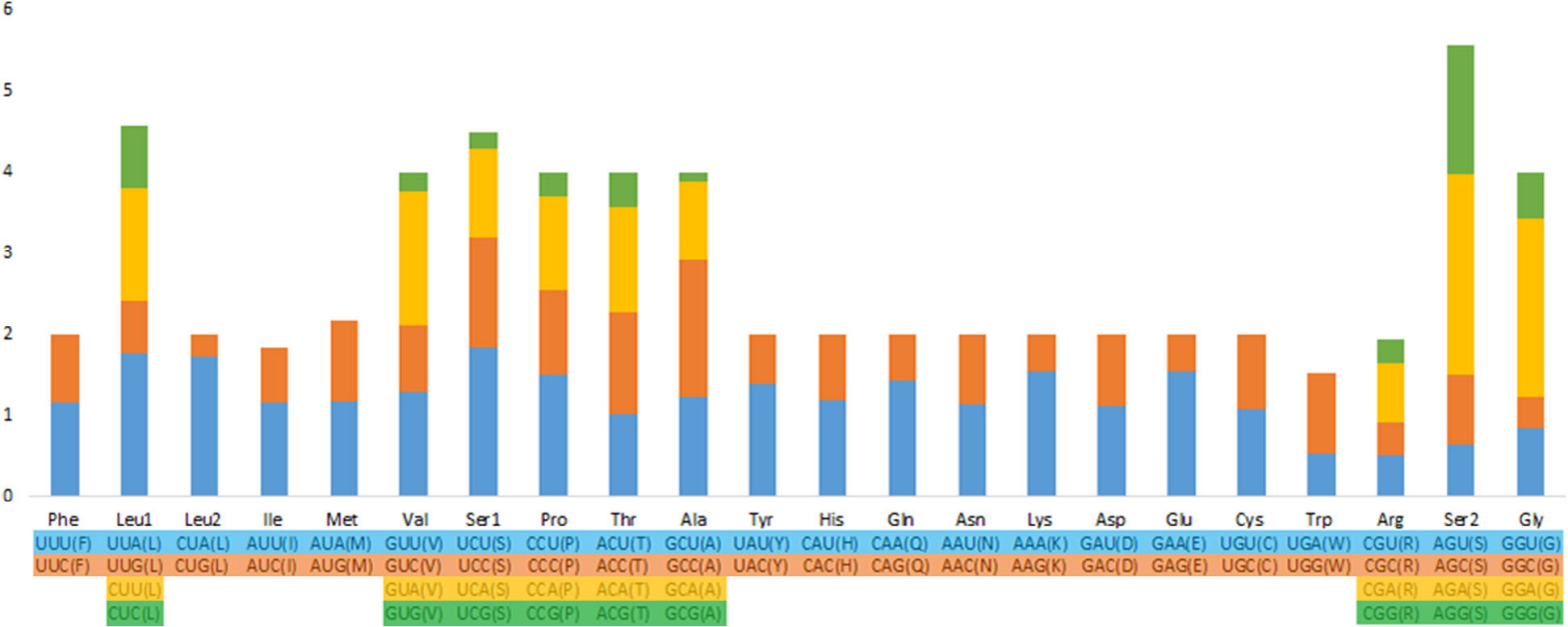
RSCU in *P. liuwutiensis* mitogenome.

Table 5 and Fig. 3 summarize the relative synonymous codon usage (RSCU) values for the 13 PCGs. The mtDNA of *P. liuwutiensis* contained 5323 codons. Among the 13 PCGs, the most frequently used amino acid was Ser (12.91%), followed by Leu (10.82%), Lys (8.34%), Pro (7.06%) and Thr (6.01%). A common feature in most metazoan mtDNA is a bias toward a higher representation of nucleotides A and T, leading to a subsequent bias in the corresponding encoded amino acids [8, 24]. The content of A + T in the 13 PCGs was 64.3% and the AT‐skew was positive, indicating a higher occurrence of A than T (Table 4).

**Table 5:** The codon number and RSCU in *P. liuwutiensis* mitochondrial protein‐coding genes.

Codon	Count	RSCU	Codon	Count	RSCU	Codon	Count	RSCU	Codon	Count	RSCU
UUU(F)	171	1.14	UCU(S)	162	1.83	UAU(Y)	212	1.39	UGU(C)	30	1.07
UUC(F)	130	0.86	UCC(S)	121	1.36	UAC(Y)	92	0.61	UGC(C)	26	0.93
UUA(L)	169	1.76	UCA(S)	97	1.09	UAA(*)	244	1.71	UGA(W)	74	0.52
UUG(L)	61	0.64	UCG(S)	18	0.20	UAG(*)	111	0.78	UGG(W)	32	1.00
CUU(L)	134	1.40	CCU(P)	141	1.50	CAU(H)	96	1.19	CGU(R)	19	0.50
CUC(L)	73	0.76	CCC(P)	99	1.05	CAC(H)	65	0.81	CGC(R)	16	0.42
CUA(L)	112	1.17	CCA(P)	107	1.14	CAA(Q)	151	1.43	CGA(R)	27	0.71
CUG(L)	27	0.28	CCG(P)	29	0.31	CAG(Q)	60	0.57	CGG(R)	12	0.31
AUU(I)	124	1.15	ACU(T)	81	1.01	AAU(N)	153	1.13	AGU(S)	57	0.64
AUC(I)	74	0.69	ACC(T)	101	1.26	AAC(N)	118	0.87	AGC(S)	77	0.87
AUA(M)	126	1.17	ACA(T)	104	1.30	AAA(K)	340	1.53	AGA(S)	94	2.46
AUG(M)	71	1.00	ACG(T)	34	0.43	AAG(K)	104	0.47	AGG(S)	61	1.60
GUU(V)	43	1.29	GCU(A)	40	1.23	GAU(D)	76	1.11	GGU(G)	25	0.83
GUC(V)	27	0.81	GCC(A)	55	1.69	GAC(D)	61	0.89	GGC(G)	12	0.40
GUA(V)	55	1.65	GCA(A)	31	0.95	GAA(E)	127	1.53	GGA(G)	66	2.20
GUG(V)	8	0.24	GCG(A)	4	0.12	GAG(E)	39	0.47	GGG(G)	17	0.57

### rRNA and tRNA genes

**Fig. 4:**
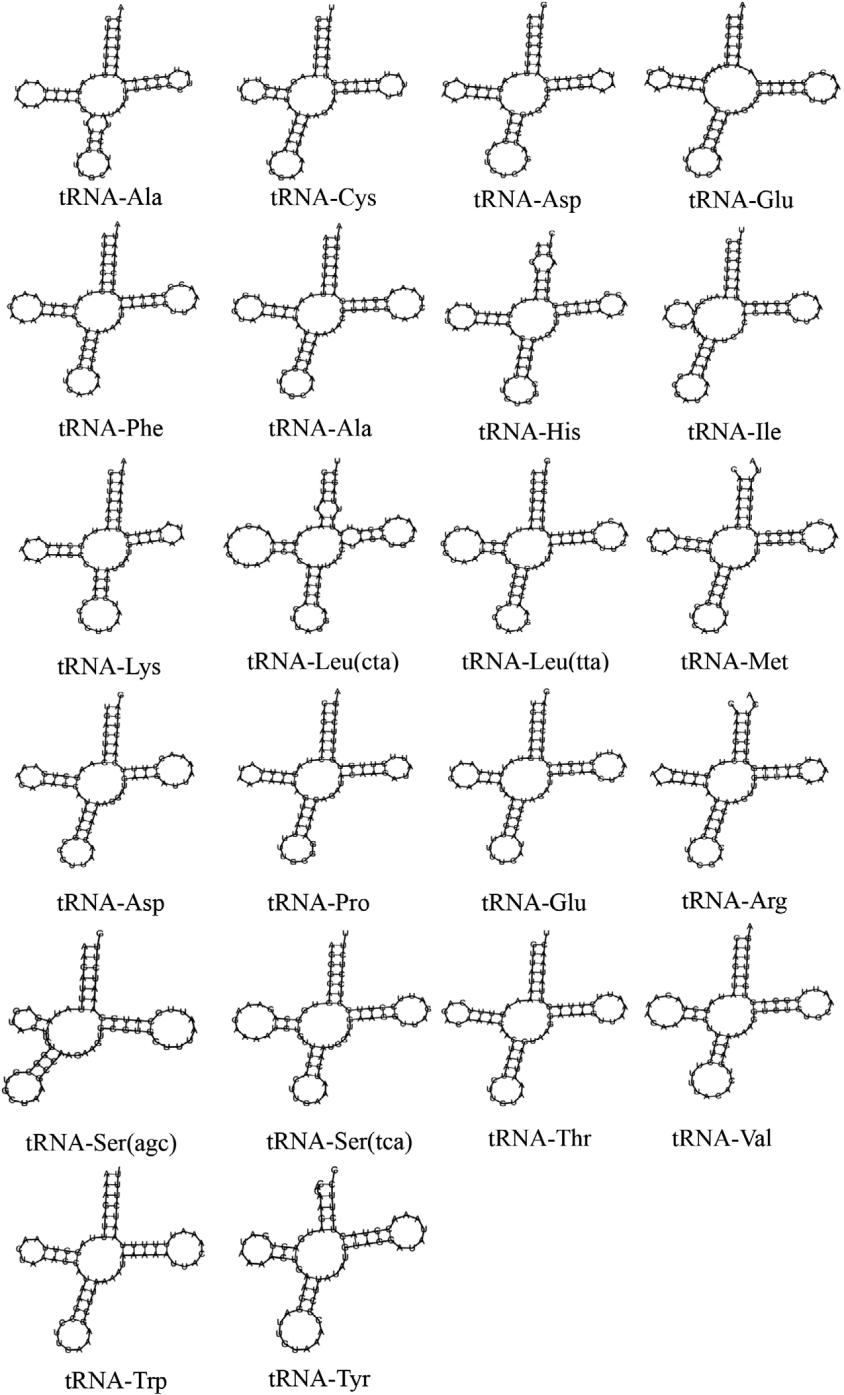
Secondary structures of the 22 tRNA genes of *P. liuwutiensis*.

The results revealed that the two rRNA of *P. liuwutiensis* were encoded on the positive strand, and the *16S* and *12S* genes were 1348 bp (G + C% = 33.16%) and 801 bp (G + C% = 37.2%) in length, respectively (Tables 3 and 4). The *16S* gene was between *nad2* and *cox1*, and *12S* was between *trnF* and *trnE*. The results showed that 5/22 tRNA genes, including *trna‐gln*, *trna‐ala*, *trna‐ser (tga)*, *trna‐asp* and *trna‐val*, were encoded on the negative strand, while the remaining genes were encoded on the positive strand. The length of the tRNA genes ranged from 64 to 73 bp, and the shortest (64 bp) and longest (73 bp) lengths were found from *trna‐lys* and *trna‐leu^(tag)^*, respectively (Table 3). In addition, Table 4 shows the base composition of tRNA A + T bias. All 22 tRNA genes were predicted to be capable of folding into a cloverleaf secondary structure using the MITOS web server (Fig. 4).

### Phylogenetic analysis

**Fig. 5:**
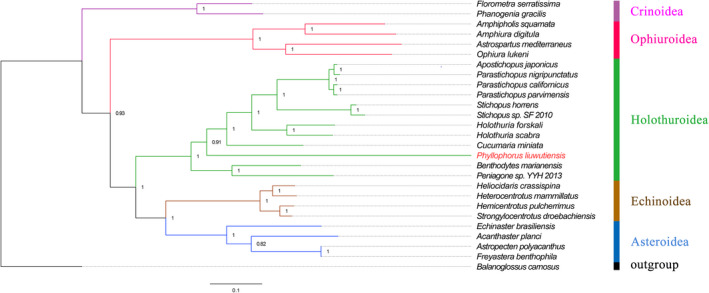
Phylogenetic trees based on the concatenated amino acid of 13 PCGs. The *Balanoglossus carnosus* (NC001887.1) is used as outgroup. The red name highlights the species sequenced in this study. *P. liuwutiensis* (MN198190), *Amphiura digitula* (MH791160.1), *Apostichopus japonicus* (FJ986223.1), *P. nigripunctatus* (AB525762.1), *Parastichopus parvimensis* (KU168761.1), *Parastichopus californicus* (KP398509.1), *Stichopus* sp. SF‐2010 (HM853683.2), *Stichopus horrens* (HQ000092.1), *Holothuria scabra* (KP257577.1), *Holothuria forskali* (FN562582.1), *Cucumaria miniata* (AY182376.1), *Benthodytes marianensis* (MH208310.1), *Freyastera benthophila* (MG563681.1), *Astropecten polyacanthus* (AB183560.1), *Acanthaster planci* (AB231475.1), *Echinaster brasiliensis* (MG636999.1), *Heliocidaris crassispina* (KC479025.1), *Heterocentrotus mammillatus* (KJ680292.1), *Strongylocentrotus droebachiensis* (EU054306.1), *Hemicentrotus pulcherrimus* (KC490911.1), *Florometra serratissima* (NC001878.1), *Phanogenia gracilis* (DQ068952.1), *Ophiura lutkeni* (AY184223.1), *Peniagone* sp. YYH‐2013 (KF915304.1), *Amphipholis squamata* (FN562578.1) and *Astrospartus mediterraneus* (NC013878.1).

Phylogenetic analysis revealed that the complete mtDNA of *P. liuwutiensis* and the other 25 echinoderm species were separated into five major clades as follows: Holothuroidea, Echinoidea, Asteroidea, Crinoidea and Ophiuroidea (Fig. 5). Almost all of the clades were strongly supported. The two species, *P. liuwutiensis* and *Cucumaria miniata*, formed a cluster, which were distinguished from other species, indicating that these two species together belonged to the same order (Dendrochirotida).

### Gene order

**Fig. 6:**
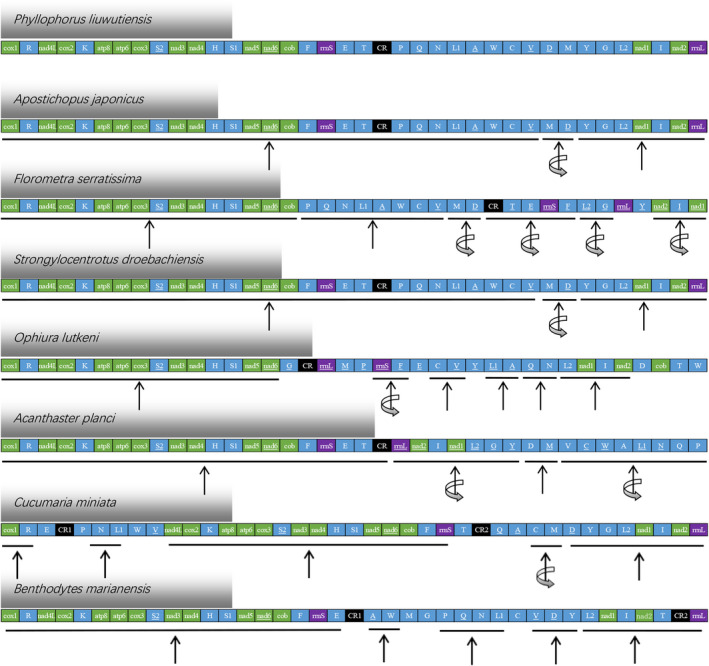
Linear representation of gene rearrangements of *P. liuwutiensis*, *Apostichopus japonicus*, *Florometra serratissima*, *Strongylocentrotus droebachiensis*, *Ophiura lutkeni*, *Acanthaster planci*, *Cucumaria miniata* and *Benthodytes marianensis*. Gene segments are not drawn to scale. All genes are transcribed from left to right except those indicated by underlining, which are transcribed from right to left. The circling arrows indicate inversions. tRNA genes are represented by the corresponding single‐letter amino acid code, especially S1 (AGN), S2 (UCN), L1 (CUN) and L2 (UUR). *rrnL* and *rrnS* are the large and small rRNA subunits, respectively.

Figure 6 illustrates the gene arrangement of *P. liuwutiensis* and seven other echinoderms. Similar gene arrangements exist in the species used to construct evolutionary trees or that had a few gene rearrangements. *Holothuria scabra*, *Holothuria forskali*, *Apostichopus japonicus*, *Parastichopus nigripunctatus*, *Parastichopus parvimensis* and *Parastichopus californicus* had the same gene arrangements [3]. *Florometra serratissima* and *P. gracilis* also had the same gene arrangement. The gene arrangement of *Astropecten polyacanthus* and *A. planci* is the same [9]. The gene order of *A. japonicus* (class Holothuroidea), *S. droebachiensis* (class Echinoidea) and *P. liuwutiensis* had similar changes with the inversion occurring between the genes *trnD* and *trnM* (Fig. 6), including the two conserved genetic blocks (*cox1‐R‐nad4L‐cox2‐K‐atp8‐atp6‐cox3‐S2‐nad3‐nad4‐H‐S1‐nad5‐nad6‐cob‐F‐rrnS‐E‐T‐P‐Q‐N* and *Y‐G‐L2‐nad1‐I‐nad2‐rrnL‐L1‐A‐W‐C‐V*), which were found in the same order. The gene order of *F. serratissima* (class Crinoidea) was significantly different from that of *P. liuwutiensis*, with more translocations and inversions, and two genes were rearranged (*rrnL* and *V*). *Ophiura lutkeni* (class Ophiuroidea) produced more translocations. Therefore, these 10 genes (*G*, *rrnL*, *M*, *P*, *E*, *Y*, *D*, *cob*, *T* and *W*) were involved in the gene rearrangement, and five conserved gene blocks (*cox1‐R‐nad4L‐cox2‐K‐atp8‐atp6‐cox3‐S2‐nad3‐nad4‐H‐S1‐nad5‐nad6*, *C‐V*, *L1‐A*, *Q‐N* and *L2‐nad1‐I‐nad2*) were found in the same order. In contrast, *A. planci* (class Asteroidea) had two parts of a wide range of gene location inversions (*trnY*‐*rrnL* and *trnP‐trnV*), and the translocations of these two parts were observed. The only two conserved genetic blocks (*cox1‐R‐nad4L‐cox2‐K‐atp8‐atp6‐cox3‐S2‐nad3‐nad4‐H‐S1‐nad5‐nad6‐cob‐F‐rrnS‐E‐T* and *D‐M*) were found in the same order. Four conserved gene blocks were found (*cox1‐R*, *N‐L1*, *nad4L‐cox2‐K‐atp8‐atp6‐cox3‐S2‐nad3‐nad4‐H‐S1‐nad5‐nad6‐cob‐F‐rrnS*, *Y‐G‐L2‐nad1‐I‐nad2‐rrnL*) in the gene arrangement of *C. miniata* (class Holothuroidea), and the inversion occurred between the genes *trnD* and *trnM*. In the sea cucumber *Benthodytes marianensis* (class Holothuroidea), five conserved gene blocks (*atp6‐cox3‐S2‐nad3‐nad4‐H‐S1‐nad5‐nad6‐cob‐F‐rrnS‐E*, *A‐W*, *P‐Q‐N‐L1*, *C‐V‐D*, *L2‐nad1‐I‐nad2‐T‐rrnL*) were identified. Meanwhile, compared with the gene arrangement of *P. liuwutiensis*, the translocation of three tRNAs (*M*, *G* and *Y*) can be found.

## Discussion

Phylogenetics studies the evolutionary relationships of organisms, and the phylogenetic tree is the topological structure that describes the evolutionary order of various groups of organisms [25, 26]. However, with the development and application of molecular biology, our understanding of genes and proteins has been constantly increasing, and a theoretical method has been gradually developed to study the evolutionary relationship of species based on the genetic information of biological macromolecules, such as DNA or protein sequence. Because most of the mitochondrial genes are matrilineal and the sequence variation is rapid, they are widely used to study the phylogenetic evolution [27, 28]. The most basic problem solved by phylogenetic research is to determine the taxonomic status of species [29, 30]. However, determining the relationship of species can further infer the biological characteristics of unknown species, which, of course, requires a lot of phylogenetic studies to prove [31]. Each newly sequenced species enriches the database, providing more information for studying the phylogeny between species.

In this study, we conducted a preliminary study on the mtDNA of *P. liuwutiensis*. Mitochondrial genomes are maternal mtDNA and do not recombine DNA. Therefore, individuals with the same mtDNA sequence are descended from the same female ancestor [32]. Consequently, mtDNA sequences can be used to determine the relationships between species. In this study, we aim to characterize the mtDNA of *P. liuwutiensis*, as well as to determine the taxonomic relationship between *P. liuwutiensis* and other echinoderm species.

The results showed that the contents of base A and base T were higher, the content of A + T was higher compared with G + C, and the lowest content of A + T was 56.34% (*A. planci*), indicating the characteristics of mtDNA sequence in invertebrates [33]. The CR was found to be the main noncoding region of the mtDNA, which is necessary for the initiation of mtDNA transcription and replication of metazoa [34, 35]. Its size and nucleotide sequence greatly varied. Most metazoa mtDNA contain only one CR, whereas in some sea cucumbers there are two duplicate CRs or two independent CRs with the same or highly similar nucleotide sequences [33]. The mtDNA of *P. liuwutiensis* was found to contain a CR between *tRNA^thr^* and *tRNA^pro^*. Three mechanisms may contribute to mtDNA with duplicate CRs: tandem duplication, dimerization and illegitimate recombination. Many studies support the idea that mitogenome with repetitive CRs may replicate more efficiently than mitogenome with a single CR [36, 37]. However, because only two species of sea cucumber (*B. marianensis* and *Cucumaria miniate*) have been shown to have duplicate CRs, additional mitotic genomes are needed to elucidate the mechanism that causes this phenomenon [3].

Based on the basic assumption that shared genetic arrangements imply a common ancestor, it is highly unlikely that the same sequence of genes will emerge independently in different lineages [9]. Therefore, comparative gene alignment may be a useful tool for phylogenetic studies, especially when some ancestral relationships are concerned. Over the past two decades, many studies have been reported on the mitochondrial gene sequence in echinoderms [38, 39]. There are four possible mechanisms for genome rearrangement: inversion (reversals), transposition, reverse transposition and tandem duplication random losses [3]. It is of great significance to explore the evolutionary history of mitochondrial gene rearrangement in echinoderms by comparing the gene arrangement in the mtDNA of echinoderms and studying the common sequence among different individuals [40, 41]. In this study, the gene arrangement of *P. liuwutiensis* was very similar to that of the other five echinoderm species, which all contained conserved gene blocks of 15 genes. In particular, *A. japonicus* and *S. droebachiensis* had an inversion of only two genes compared with *P. liuwutiensis*. Shen *et al*. [9] have hypothesized that the inclusion of a consensus nonavian vertebrate gene order does support the echinoid mtDNA gene order as the most likely representative of the echinoderm ground pattern. Genes and the environment act together on biological traits, with genes playing a dominant role. Therefore, the study of genes can infer the adaptability of species to the environment. Mu *et al*. [3] have studied the adaptation of sea cucumber to the deep‐sea environment through the mtDNA of sea cucumber, and predicted that *nad2* and *nad4* might be important candidate genes for the further study on the adaptation of *B. marianensis* to the deep‐sea environment. The different gene order may be related to certain ecological or morphological features of the species. Genes may produce different effects in different positions, and the interactions between genes have different results. These assumptions need to be tested by further research.

## Conclusions

In this study, we characterized the structure of the mtDNA of *P. liuwutiensis*, and the results showed that the mtDNA (15 969 bp) encoded 37 genes, including 13 PCGs, 22 tRNA genes and 2 rRNA genes. One putative CR was found in the mitogenome of *P. liuwutiensis*. The mtDNA of *P. liuwutiensis* was clustered together with *C. miniate*. Moreover, the gene arrangement of *P. liuwutiensis* was also described in detail.

## Conflict of interest

The authors declare no conflict of interest.

## Author contributions

QY and QL designed and supervised the research. FY performed most of the experiments and wrote the paper with assistance from C. Zhou, NTT, ZS, JW, HG, ZL, C. Zhong and ZZ. All authors made contributions to the final version of this manuscript. All authors read and approved the final manuscript.

## Data Availability

All original sequence data in this study were submitted to the NCBI database under accession number MN198190.
